# Synthesis and crystal structures of *N*-H, *N*-phenyl and *N*-benzyl-2-(4-hexyl­oxyphen­yl)benzimidazoles

**DOI:** 10.1107/S2056989021004898

**Published:** 2021-05-14

**Authors:** Daniil E. Smirnov, Sergei V. Tatarin, Stanislav I. Bezzubov

**Affiliations:** a Department of Chemistry, Lomonosov Moscow State University, Lenin’s Hills, 1-3, Moscow, 119991, Russian Federation; b N. S. Kurnakov Institute of General and Inorganic Chemistry, Russian, Academy of Sciences, Leninsky pr. 31, Moscow 119991, Russian Federation

**Keywords:** crystal structure, benzimidazole, synthesis, H-bonding, NMR study

## Abstract

The title compounds, 2-(4-hexyl­oxyphen­yl)-1*H*-benzimidazole (C_19_H_22_N_2_O; **1**), 2-(4-hexyl­oxyphen­yl)-1-phenyl-1*H*-benzimidazole (C_25_H_26_N_2_O; **2**) and 1-benzyl-2-(4-hexyl­oxyphen­yl)-1*H*-benzimidazole (C_26_H_28_N_2_O; **3**) were synthesized and their structures were determined by single-crystal X-ray analysis. The N-substituent at the imidazole moiety slightly affects the inter­planar angle between the 4-hexyl­oxyphenyl ring and the benzimidazole system. The unsubstituted benzimidazole (**1**) forms inter­molecular N—H⋯N bonds while in the crystal structures of **2** and **3**, the mol­ecules are assembled only through π–π and C—H⋯π inter­actions.

## Abstract 2

*N*-H, *N*-phenyl and *N*-benzyl-2-(4-hexyl­oxyphen­yl)benzimidazoles were prepared and studied by ^1^H NMR and single-crystal X-ray analysis. The unsubstituted benzimidazole forms inter­molecular N—H⋯N bonds while in the crystal structures of the other two compounds, the mol­ecules are assembled only through π–π and C—H⋯π inter­actions.

## Chemical context   

2-Aryl­benzimidazoles have attracted considerable attention as biologically active compounds (Vasava *et al.*, 2020 ▸). They are also used as ligands in constructing cyclo­metalated iridium(III) and ruthenium(II) complexes for organic light-emitting diodes and photosensitizers in dye-sensitized solar cells (Bezzubov *et al.*, 2020 ▸; Lavrova *et al.*, 2020 ▸). For the latter application, the aryl unit of these ligands should contain π-electron-donating substituents to increase the light-harvesting characteristics of the corresponding organometallic complexes (Aghazada & Nazeeruddin, 2018 ▸; Bezzubov *et al.*, 2014 ▸, 2016 ▸). In addition, long aliphatic chains in the ligands are preferable to diminish aggregation of the complexes on the semiconductor surface (Hagfeldt *et al.*, 2010 ▸). In line with this, we synthesized 2-(4-hexyl­oxyphen­yl)-1*H*-benzimidazole (**1**) and its *N*-phenyl and *N*-benzyl analogues (**2** and **3**, respectively) and studied their crystal structures.
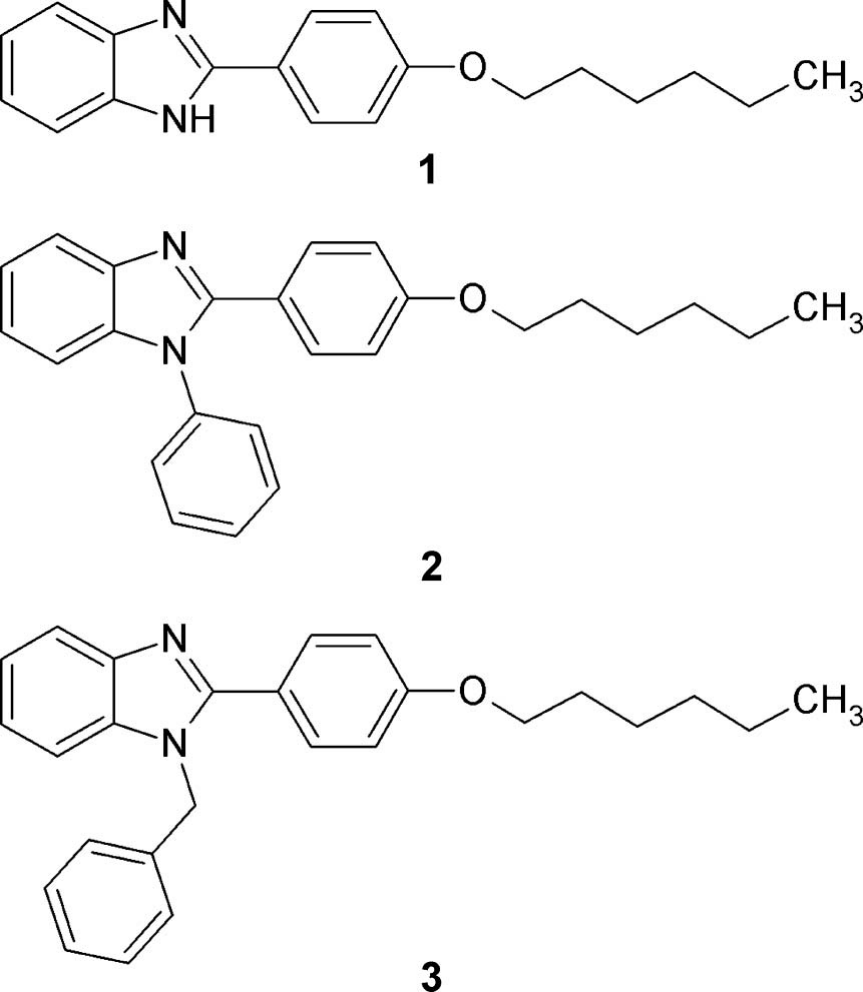


## Structural commentary   

**Figure 1:**
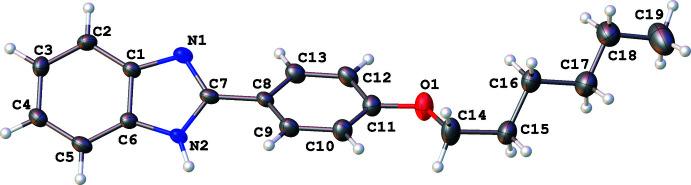
The mol­ecular structure of 2-(4-hexyl­oxyphen­yl)-1*H*-benzim­id­azole (**1**), with displacement ellipsoids drawn at the 50% probability level.

In all three structures, the organic mol­ecules occupy general positions and contain identical benzimidazole and 4-hexyl­oxyphenyl units and different N-substituents (Figs. 1 ▸–3 ▸ ▸). The benzimidazole systems are essentially flat while the alkoxyaryl rings are inclined to them with dihedral angles of 35.02 (17), 31.46 (4) and 38.67 (6)° for **1**, **2** and **3**, respectively. Although the *N*-phenyl ring is expected to exert a larger steric pressure in **2** as compared with **3**, its rotation by 68.92 (4)° along the N2—C8 bond seems to reduce the steric hindrance in the mol­ecule and results in the smallest inter­planar angle between the aryl and imidazole moieties in the series. In the structures of **1** and **2**, the hydro­carbon chains crystallize in the common *trans* zigzag conformation, while in the structure of **3** the chain adopts a *gauche* conformation about the C23–C24 bond.

In the ^1^H NMR spectra of **1**–**3**, a similar set of high-field multiplets assigned to protons of the aliphatic chain was observed. In contrast, the NMR pattern corresponding to the aromatic protons in the substances becomes more complex when going from **1** to **2** and **3**. In the aromatic part of ^1^H NMR spectrum of **1**, there are four individual resolved multiplets corresponding to the symmetric benzimidazole part, assuming rapid exchange of the N–H proton on the NMR time scale. Phenyl or benzyl substituents at the nitro­gen atom decrease the symmetry of the benzimidazole moiety, which results in the appearance of additional signals that are highly overlapped and make the spectra of **2** and **3** difficult to inter­pret.

## Supra­molecular features   

**Figure 4:**
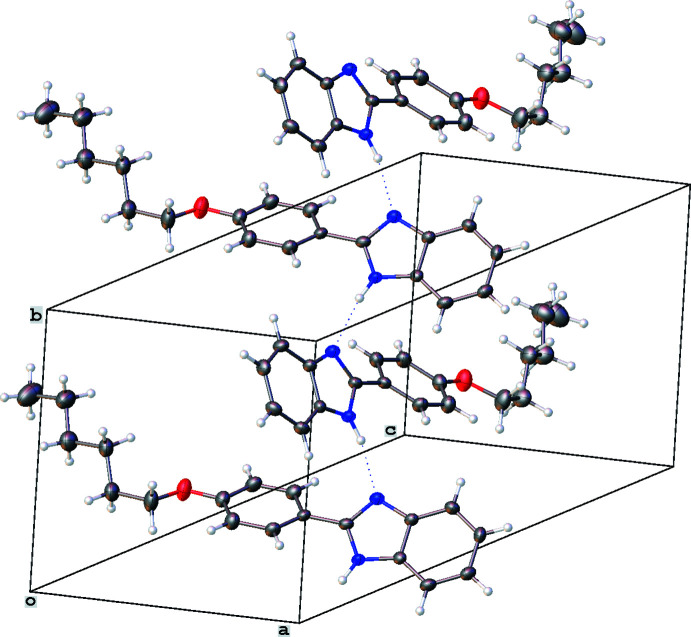
Hydrogen bonding in the crystal of 2-(4-hexyl­oxyphen­yl)-1*H*-benzim­id­azole (**1**). Displacement ellipsoids are shown at the 50% probability level.

**Figure 5:**
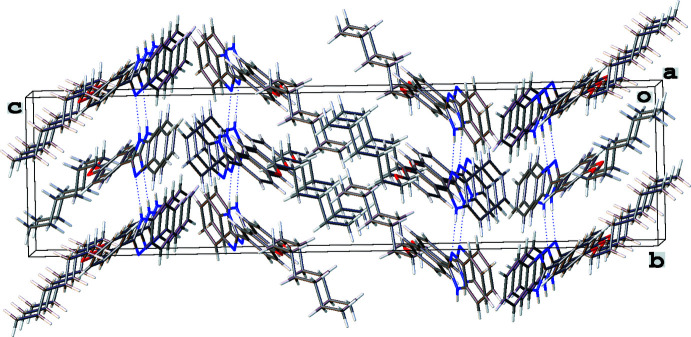
Fragment of the crystal packing of 2-(4-hexyl­oxyphen­yl)-1*H*-benzimidazole (**1**).

In the crystal of **1**, mol­ecules related by the *b* glide plane are assembled through N—H⋯N bonds (Fig. 4 ▸, Table 1 ▸). The resulting chains are grafted together in a herringbone-like manner by C—H⋯π inter­actions between the H3 atom and the N1/C1/C6/N2/C7_centroid_ [3.025 (18) Å, 126.2 (3)°] and between the H10 atom and the C1–C6_centroid_ [3.245 (18) Å, 142.7 (3)°]. Along the *c* axis, these relatively dense crystal subunits alternate with less dense regions filled by aliphatic chains held together only *via* van der Waals inter­actions (Fig. 5 ▸).

**Figure 6:**
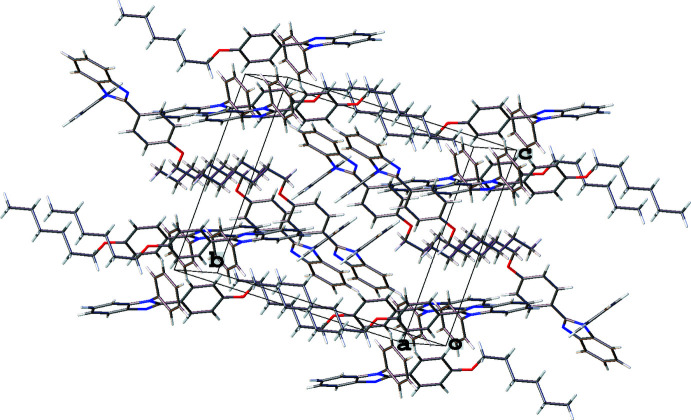
Fragment of the crystal packing of 2-(4-hexyl­oxyphen­yl)-1*-*phenyl-1*H-*benzimidazole (**2**).

In the crystal of **2** (Fig. 6 ▸), there are centrosymmetric dimers in which individual mol­ecules are joined by C—H⋯π contacts involving the H20*B* atom and the C1–C6_centroid_ [2.681 (15) Å, 178.4 (11)°] as well as the H9 atom and the C14–C19_centroid_ [2.809 (15) Å, 144.2 (10)°]. These dimers form the 3-D packing *via* van der Waals inter­actions between the alk­oxy chains.

**Figure 7:**
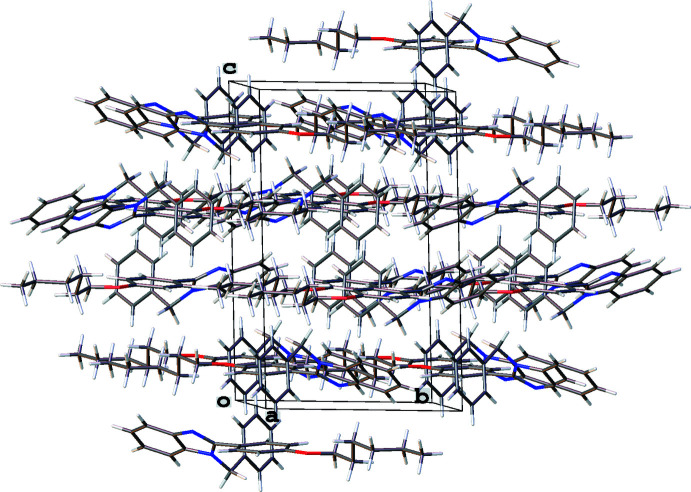
Fragment of the crystal packing of 1*-*benzyl-2-(4-hexyl­oxyphen­yl)-1*H*-benzimidazole (**3**).

In the crystal of **3** (Fig. 7 ▸), mol­ecules related by a twofold screw axis are linked *via* C—H⋯π contacts between the H8*B* atom and the centroid of the C1–C6 ring [2.695 (14) Å, 128.89 (10)°], between the H14 atom and the centroid of the N1–C7 imidazole ring [2.904 (14) Å, 137.45 (10)°] and between the H3 atom and the centroid of the C9–C14 ring [2.955 (14) Å, 141.31 (12)°]. In addition, each mol­ecule and its symmetry equivalent through the inversion center are linked by C—H⋯π contacts between the H23*B* atom and the centroid of the C1–C6 ring [2.847 (14) Å, 165.59 (13)°] and between the H21*A* atom and the centroid of the N1–C7 imidazole ring [2.973 (16) Å, 157.90 (12)°]. These inter­actions organize the mol­ecules into thick layers parallel to (10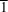) with the layers assembled by van der Waals inter­actions between the alk­oxy chains.

## Database survey   

Although the crystal structures of more than a thousand 2-aryl­benzimidazoles have been published so far (Cambridge Structural Database, version 5.42 updated to November 2020; Groom *et al.*, 2016 ▸), fewer than 20 of them (including a few metal complexes) contain eth­oxy groups or longer chains attached to the aryl ring (Geiger *et al.*, 2017 ▸; Wang, Niu *et al.*, 2014 ▸; Rahman *et al.*, 2012 ▸; Wadhwa *et al.*, 2016 ▸; Yeap *et al.*, 2009 ▸; Ha, 2012 ▸; Wang, Sun *et al.*, 2014 ▸). It is inter­esting to note that 5-(2-(*p*-chloro­phenyl­benzimidazol-1-yl-meth­yl)-4-(2-methyl­phen­yl)-2,4-di­hydro-[1,2,4]-triazole-3-thione is iso­struct­ural with compound **2** (Karayel *et al.*, 2015 ▸).

## Synthesis and crystallization   

The title compounds were prepared as follows:

**2-(4-Hexyl­oxyphen­yl)-1** ***H*** **-benzimidazole (1)**

A mixture of 1,2-di­amino­benzene (108 mg, 1 mmol), 4-(hex­yloxy)benzaldehyde (0.208 ml, 1 mmol) and sodium metabisulfite (190 mg, 1 mmol) in ethanol (30 mL) was refluxed under Ar for 3 h. The reaction mixture was evap­orated to dryness, washed with water and di­chloro­methane and the white powder was collected and dried *in vacuo*. Yield 242 mg (82%). Single crystals suitable for X-ray analysis were grown by slow evaporation of the solvent from a solution of the substance in an acetone/water mixture, m.p. = 472–473 K

^1^H NMR [(CD_3_)_2_CO, ppm, 400 MHz]: δ 8.19–8.11 (*m*, 2H, H_Ar_), 7.59–7.52 (*m*, 2H, H_Ar_), 7.22–7.13 (*m*, 2H, H_Ar_), 7.12–7.04 (*m*, 2H, H_Ar_), 4.09 (*t*, *J* = 6.5 Hz, 2H, H_Alk_), 1.86–1.75 (*m*, 2H, H_Alk_), 1.56–1.44 (*m*, 2H, H_Alk_), 1.43–1.31 (*m*, 4H, H_Alk_), 0.95–0.87 (*m*, 3H, H_Alk_).

**2-(4-Hexyl­oxyphen­yl)-1** ***-*** **phenyl-1** ***H*** **-benzimidazole (2)**

A mixture of *N*-phenyl­benzene-1,2-di­amine (1.84 g, 10 mmol), 4-(hex­yloxy)benzaldehyde (1.66 mL, 8 mmol) and sodium metabisulfite (1.9 g, 10 mmol) in ethanol (15 mL) was refluxed under Ar for 5 h. The solvent was removed *in vacuo* and the crude product was recrystallized from a water/acetone mixture. Yield 2.083 g (70%). Single crystals suitable for X-ray analysis were collected from the recrystallized product, m.p. = 389–390 K.

^1^H NMR (CDCl_3_, ppm, 400 MHz): δ 7.87 (*d*, *J* = 8.0 Hz, 1H, H_Ar_), 7.55–7.41 (*m*, 5H, H_Ar_), 7.36–7.28 (*m*, 3H, H_Ar_), 7.28–7.18 (*m*, 2H, H_Ar_), 6.85–6.77 (*m*, 2H, H_Ar_), 3.97–3.89 (*t*, *J* = 6.6 Hz, 2H, H_Alk_), 1.82–1.70 (*m*, 2H, H_Alk_), 1.50–1.40 (*m*, 2H, H_Alk_), 1.40–1.27 (m, 4H, H_Alk_), 0.96–0.87 (*m*, 3H, H_Alk_).

**1** ***-*** **Benzyl-2-(4-hexyl­oxyphen­yl)-1** ***H*** **-benzimidazole (3)**

To a suspension of **1** (160 mg, 0.542 mmol) in aceto­nitrile (30 mL), caesium carbonate (265 mg, 0.813 mmol) and benzyl bromide (0.067 mL, 0.569 mmol) were added. The reaction mixture was stirred at room temperature for 12 h and concentrated *in vacuo*. The residue was dissolved in a mixture of CH_2_Cl_2_ and sat. NaHCO_3_. The aqueous layer was extracted with CH_2_Cl_2_, the organic layers were combined and the solvent was removed *in vacuo*. Recrystallization from CH_2_Cl_2_/EtOH gave the product as a white powder. Yield 188 mg (90%). Single crystals suitable for X-ray analysis were grown by slow evaporation of the solvent from a solution of the substance in a CHCl_3_/EtOH mixture (3/1 *v*:*v*), m.p. = 401–402 K.

^1^H NMR (CDCl_3_, ppm, 400 MHz): δ 7.86 (*d*, *J* = 8.0 Hz, 1H, H_Ar_), 7.66–7.58 (*m*, 2H, H_Ar_), 7.39–7.27 (*m*, 4H, H_Ar_), 7.25–7.17 (*m*, 2H, H_Ar_), 7.15–7.10 (*m*, 2H, H_Ar_), 7.00–6.92 (*m*, 2H, H_Ar_), 5.48–5.44 (*s*, 2H, H_Ar_), 4.00 (*t*, *J* = 6.6 Hz, 2H, H_Alk_), 1.86–1.74 (*m*, 2H, H_Alk_), 1.49–1.45 (*m*, 2H, H_Alk_), 1.39–1.31 (*m*, 4H, H_Alk_), 0.95–0.86 (*m*, 3H, H_Alk_).

## Refinement   

Crystal data, data collection and structure refinement details are summarized in Table 2 ▸. In the structures of **1** and **3**, hydrogen atoms were placed in calculated positions and refined using a riding model [C—H = 0.94–0.97 Å with *U*_iso_(H) = 1.2–1.5*U*_eq_(C)]. In the structure of **1**, the N—H hydrogen atom was located from a difference electron-density map and refined using a riding model [N—H = 0.88 Å with *U*_iso_(H) = 1.2*U*_eq_(N)]. Hydrogen atoms in the structure of **2** were located from difference electron-density maps and were refined freely.

## Supplementary Material

Crystal structure: contains datablock(s) 3, 2, 1. DOI: 10.1107/S2056989021004898/mw2176sup1.cif

Structure factors: contains datablock(s) 3. DOI: 10.1107/S2056989021004898/mw21763sup2.hkl

Supporting information file. DOI: 10.1107/S2056989021004898/mw21763sup7.mol

Supporting information file. DOI: 10.1107/S2056989021004898/mw21763sup8.cml

Structure factors: contains datablock(s) 2. DOI: 10.1107/S2056989021004898/mw21762sup3.hkl

Supporting information file. DOI: 10.1107/S2056989021004898/mw21762sup6.mol

Supporting information file. DOI: 10.1107/S2056989021004898/mw21762sup9.cml

Supporting information file. DOI: 10.1107/S2056989021004898/mw21761sup10.cml

Structure factors: contains datablock(s) 1. DOI: 10.1107/S2056989021004898/mw21761sup4.hkl

Supporting information file. DOI: 10.1107/S2056989021004898/mw21761sup5.mol

CCDC references: 2079816, 2079817, 2079815

Additional supporting information:  crystallographic information; 3D view; checkCIF report

## Figures and Tables

**Figure 2:**
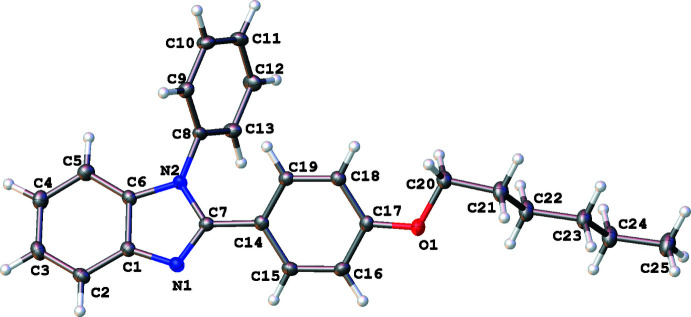
The mol­ecular structure of 2-(4-hexyl­oxyphen­yl)-1*-*phenyl-1*H-*benzimid­azole (**2**), with displacement ellipsoids drawn at the 50% probability level.

**Figure 3:**
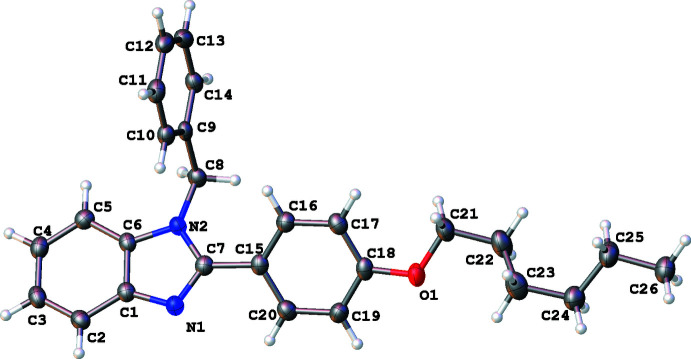
The mol­ecular structure of 1*-*benzyl-2-(4-hexyl­oxyphen­yl)-1*H-*benzimid­azole (**3**), with displacement ellipsoids drawn at the 50% probability level.

**Table 1:** Hydrogen-bond geometry (Å, °) for **1** [Chem scheme1]

*D*—H⋯*A*	*D*—H	H⋯*A*	*D*⋯*A*	*D*—H⋯*A*
N2—H2⋯N1^i^	0.88	1.99	2.861 (5)	169

Symmetry code: (i) -x+{\script{1\over 2}}, y-{\script{1\over 2}}, z.

**Table 2:** Experimental details

	**1**	**2**	**3**
Crystal data			
Chemical formula	C_19_H_22_N_2_O	C_25_H_26_N_2_O	C_26_H_28_N_2_O
*M*_r_	294.38	370.48	384.50
Crystal system, space group	Orthorhombic, *P* *b* *c* *a*	Monoclinic, *P*2_1_/*n*	Monoclinic, *P*2_1_/*n*
Temperature (K)	150	100	150
*a*, *b*, *c* (Å)	9.3802 (13), 9.4076 (13), 37.235 (5)	9.0089 (3), 16.6539 (5), 13.8400 (5)	14.3057 (13), 9.6392 (7), 16.3024 (13)
α, β, γ (°)	90, 90, 90	90, 101.419 (1), 90	90, 108.977 (3), 90
*V* (Å^3^)	3285.8 (8)	2035.36 (12)	2125.8 (3)
*Z*	8	4	4
Radiation type	Mo *K*α	Mo *K*α	Mo *K*α
μ (mm^−1^)	0.07	0.07	0.07
Crystal size (mm)	0.16 × 0.14 × 0.01	0.23 × 0.22 × 0.16	0.32 × 0.18 × 0.03

Data collection			
Diffractometer	Bruker D8 Venture	Bruker D8 Venture	Bruker D8 Venture
Absorption correction	Multi-scan (*SADABS*; Bruker, 2016 ▸)	Multi-scan (*SADABS*; Bruker, 2016 ▸)	Multi-scan (*SADABS*; Bruker, 2016 ▸)
*T*_min_, *T*_max_	0.642, 0.745	0.694, 0.746	0.694, 0.745
No. of measured, independent and observed [*I* > 2σ(*I*)] reflections	26780, 2900, 2171	22388, 6195, 4489	20533, 3813, 2875
*R*_int_	0.097	0.047	0.051
(sin θ/λ)_max_ (Å^−1^)	0.596	0.714	0.598

Refinement			
*R*[*F* ^2^ > 2σ(*F* ^2^)], *wR*(*F* ^2^), *S*	0.099, 0.233, 1.17	0.047, 0.119, 1.03	0.045, 0.116, 1.03
No. of reflections	2900	6195	3813
No. of parameters	200	357	263
H-atom treatment	H-atom parameters constrained	All H-atom parameters refined	H-atom parameters constrained
Δρ_max_, Δρ_min_ (e Å^−3^)	0.32, −0.40	0.27, −0.25	0.52, −0.25

Computer programs: *APEX3* and *SAINT* (Bruker, 2016 ▸), *SHELXT2014/5* (Sheldrick, 2015*a* ▸), *SHELXL2018/3* (Sheldrick, 2015*b* ▸) and *OLEX2* (Dolomanov *et al.*, 2009 ▸).

## supplementary crystallographic information

### 2-(4-Hexyloxyphenyl)-1H-benzimidazole (3) Crystal data

**Table:**

C_26_H_28_N_2_O	*F*(000) = 824
*M**_r_* = 384.50	*D*_x_ = 1.201 Mg m^−^^3^
Monoclinic, *P*2_1_/*n*	Mo *K*α radiation, λ = 0.71073 Å
*a* = 14.3057 (13) Å	Cell parameters from 5668 reflections
*b* = 9.6392 (7) Å	θ = 2.3–26.1°
*c* = 16.3024 (13) Å	µ = 0.07 mm^−^^1^
β = 108.977 (3)°	*T* = 150 K
*V* = 2125.8 (3) Å^3^	Plate, colourless
*Z* = 4	0.32 × 0.18 × 0.03 mm

### 2-(4-Hexyloxyphenyl)-1H-benzimidazole (3) Data collection

**Table:**

Bruker D8 Venture diffractometer	3813 independent reflections
Radiation source: microfocus sealed X-ray tube, Incoatec IµS microsource	2875 reflections with *I* > 2σ(*I*)
Focusing mirrors monochromator	*R*_int_ = 0.051
Detector resolution: 10.4 pixels mm^-1^	θ_max_ = 25.2°, θ_min_ = 2.3°
ω–scan	*h* = −17→17
Absorption correction: multi-scan (SADABS; Bruker, 2016)	*k* = −11→11
*T*_min_ = 0.694, *T*_max_ = 0.745	*l* = −19→19
20533 measured reflections	

### 2-(4-Hexyloxyphenyl)-1H-benzimidazole (3) Refinement

**Table:**

Refinement on *F*^2^	Primary atom site location: dual
Least-squares matrix: full	Hydrogen site location: inferred from neighbouring sites
*R*[*F*^2^ > 2σ(*F*^2^)] = 0.045	H-atom parameters constrained
*wR*(*F*^2^) = 0.116	*w* = 1/[σ^2^(*F*_o_^2^) + (0.0499*P*)^2^ + 0.8451*P*] where *P* = (*F*_o_^2^ + 2*F*_c_^2^)/3
*S* = 1.03	(Δ/σ)_max_ < 0.001
3813 reflections	Δρ_max_ = 0.52 e Å^−^^3^
263 parameters	Δρ_min_ = −0.25 e Å^−^^3^
0 restraints	

### 2-(4-Hexyloxyphenyl)-1H-benzimidazole (3) Special details

**Table:**

Geometry. All esds (except the esd in the dihedral angle between two l.s. planes) are estimated using the full covariance matrix. The cell esds are taken into account individually in the estimation of esds in distances, angles and torsion angles; correlations between esds in cell parameters are only used when they are defined by crystal symmetry. An approximate (isotropic) treatment of cell esds is used for estimating esds involving l.s. planes.

### 2-(4-Hexyloxyphenyl)-1H-benzimidazole (3) Fractional atomic coordinates and isotropic or equivalent isotropic displacement parameters (Å^2^)

**Table:**

	*x*	*y*	*z*	*U*_iso_*/*U*_eq_	
O1	0.35057 (10)	0.68007 (13)	0.63440 (8)	0.0346 (3)	
N2	0.71150 (11)	0.25231 (14)	0.65132 (9)	0.0245 (3)	
N1	0.57463 (11)	0.13503 (15)	0.57646 (9)	0.0265 (3)	
C7	0.61008 (13)	0.25349 (18)	0.61454 (11)	0.0251 (4)	
C9	0.83510 (13)	0.43825 (17)	0.65779 (11)	0.0239 (4)	
C6	0.74261 (13)	0.12185 (17)	0.63539 (11)	0.0241 (4)	
C15	0.54652 (13)	0.37098 (18)	0.61908 (11)	0.0254 (4)	
C1	0.65666 (13)	0.05056 (18)	0.58863 (11)	0.0251 (4)	
C17	0.50564 (14)	0.61502 (19)	0.61353 (11)	0.0289 (4)	
H17	0.522454	0.708718	0.606838	0.035*	
C18	0.41772 (13)	0.58396 (19)	0.62720 (11)	0.0281 (4)	
C8	0.77685 (13)	0.35755 (18)	0.70438 (11)	0.0265 (4)	
H8A	0.823789	0.311896	0.755747	0.032*	
H8B	0.736704	0.423506	0.725444	0.032*	
C5	0.83572 (13)	0.06191 (19)	0.65827 (11)	0.0272 (4)	
H5	0.893481	0.111446	0.690252	0.033*	
C19	0.39190 (14)	0.44582 (19)	0.63417 (12)	0.0307 (4)	
H19	0.330604	0.424005	0.642021	0.037*	
C2	0.66237 (14)	−0.08558 (18)	0.56151 (12)	0.0287 (4)	
H2	0.604818	−0.134800	0.528710	0.034*	
C14	0.91141 (13)	0.52387 (18)	0.70620 (12)	0.0287 (4)	
H14	0.925754	0.530467	0.767205	0.034*	
C10	0.81481 (14)	0.43126 (18)	0.56852 (11)	0.0278 (4)	
H10	0.762893	0.373364	0.534517	0.033*	
C20	0.45566 (14)	0.34143 (19)	0.62962 (11)	0.0298 (4)	
H20	0.437451	0.247555	0.633726	0.036*	
C16	0.56943 (14)	0.50850 (18)	0.60961 (11)	0.0275 (4)	
H16	0.629935	0.530316	0.600256	0.033*	
C4	0.84007 (14)	−0.07356 (19)	0.63211 (12)	0.0303 (4)	
H4	0.902394	−0.118716	0.646990	0.036*	
C3	0.75487 (15)	−0.14613 (19)	0.58417 (12)	0.0315 (4)	
H3	0.760861	−0.238922	0.566880	0.038*	
C11	0.86981 (14)	0.50819 (19)	0.52880 (12)	0.0322 (5)	
H11	0.854981	0.503161	0.467686	0.039*	
C13	0.96659 (14)	0.59952 (19)	0.66617 (13)	0.0327 (5)	
H13	1.018967	0.656944	0.699922	0.039*	
C12	0.94587 (14)	0.59196 (19)	0.57721 (13)	0.0337 (5)	
H12	0.983728	0.644025	0.549780	0.040*	
C21	0.37783 (15)	0.82380 (19)	0.63491 (13)	0.0363 (5)	
H21A	0.380986	0.851037	0.577313	0.044*	
H21B	0.443518	0.839772	0.678704	0.044*	
C24	0.12096 (16)	0.9823 (2)	0.61640 (14)	0.0414 (5)	
H24A	0.054475	0.959653	0.575868	0.050*	
H24B	0.121849	0.957698	0.675606	0.050*	
C26	0.05543 (16)	1.2226 (2)	0.62688 (14)	0.0416 (5)	
H26A	−0.007303	1.199254	0.582282	0.062*	
H26B	0.069379	1.321545	0.623109	0.062*	
H26C	0.050857	1.202249	0.684342	0.062*	
C25	0.13774 (16)	1.1376 (2)	0.61283 (15)	0.0445 (6)	
H25A	0.200929	1.162309	0.657797	0.053*	
H25B	0.143581	1.161314	0.555610	0.053*	
C23	0.19767 (16)	0.8929 (2)	0.59333 (14)	0.0448 (5)	
H23A	0.177602	0.794288	0.591145	0.054*	
H23B	0.198534	0.919180	0.534875	0.054*	
C22	0.29974 (16)	0.9082 (2)	0.65685 (14)	0.0466 (6)	
H22A	0.298728	0.880129	0.714969	0.056*	
H22B	0.318687	1.007348	0.660047	0.056*	

### 2-(4-Hexyloxyphenyl)-1H-benzimidazole (3) Atomic displacement parameters (Å^2^)

**Table:**

	*U*^11^	*U*^22^	*U*^33^	*U*^12^	*U*^13^	*U*^23^
O1	0.0335 (8)	0.0275 (7)	0.0420 (8)	0.0087 (6)	0.0113 (6)	−0.0028 (6)
N2	0.0262 (9)	0.0221 (8)	0.0247 (8)	0.0010 (6)	0.0075 (7)	−0.0020 (6)
N1	0.0269 (8)	0.0252 (8)	0.0264 (8)	0.0023 (7)	0.0073 (7)	−0.0011 (6)
C7	0.0275 (10)	0.0271 (10)	0.0208 (9)	0.0028 (8)	0.0079 (8)	0.0027 (7)
C9	0.0249 (10)	0.0198 (9)	0.0266 (9)	0.0057 (7)	0.0079 (8)	−0.0014 (7)
C6	0.0305 (10)	0.0218 (9)	0.0221 (9)	0.0025 (8)	0.0112 (8)	0.0019 (7)
C15	0.0277 (10)	0.0270 (10)	0.0199 (9)	0.0044 (8)	0.0056 (8)	−0.0006 (7)
C1	0.0289 (10)	0.0247 (9)	0.0223 (9)	0.0021 (8)	0.0093 (8)	0.0018 (7)
C17	0.0356 (11)	0.0243 (10)	0.0252 (9)	0.0028 (8)	0.0078 (8)	0.0011 (7)
C18	0.0299 (10)	0.0283 (10)	0.0229 (9)	0.0086 (8)	0.0042 (8)	−0.0004 (7)
C8	0.0280 (10)	0.0260 (9)	0.0240 (9)	0.0004 (8)	0.0061 (8)	−0.0030 (7)
C5	0.0276 (10)	0.0300 (10)	0.0239 (9)	0.0008 (8)	0.0083 (8)	0.0027 (8)
C19	0.0264 (10)	0.0311 (11)	0.0347 (11)	0.0019 (8)	0.0100 (9)	−0.0017 (8)
C2	0.0344 (11)	0.0248 (10)	0.0285 (10)	−0.0013 (8)	0.0125 (8)	0.0002 (8)
C14	0.0306 (11)	0.0245 (9)	0.0285 (10)	0.0041 (8)	0.0064 (8)	−0.0024 (8)
C10	0.0277 (10)	0.0269 (10)	0.0274 (10)	0.0049 (8)	0.0069 (8)	−0.0009 (8)
C20	0.0325 (11)	0.0253 (10)	0.0294 (10)	0.0008 (8)	0.0068 (8)	0.0002 (8)
C16	0.0290 (10)	0.0291 (10)	0.0248 (10)	0.0025 (8)	0.0093 (8)	0.0007 (8)
C4	0.0321 (11)	0.0305 (10)	0.0309 (10)	0.0076 (9)	0.0138 (9)	0.0047 (8)
C3	0.0418 (12)	0.0234 (10)	0.0339 (10)	0.0039 (9)	0.0186 (9)	0.0010 (8)
C11	0.0376 (12)	0.0318 (11)	0.0303 (10)	0.0088 (9)	0.0153 (9)	0.0039 (8)
C13	0.0282 (11)	0.0243 (10)	0.0439 (12)	−0.0001 (8)	0.0093 (9)	−0.0020 (8)
C12	0.0333 (11)	0.0259 (10)	0.0463 (12)	0.0049 (9)	0.0190 (10)	0.0068 (9)
C21	0.0405 (13)	0.0259 (11)	0.0420 (12)	0.0063 (9)	0.0126 (10)	−0.0006 (8)
C24	0.0395 (13)	0.0363 (12)	0.0483 (13)	0.0073 (10)	0.0144 (10)	0.0030 (10)
C26	0.0441 (13)	0.0420 (12)	0.0421 (12)	0.0102 (10)	0.0188 (10)	0.0014 (10)
C25	0.0457 (14)	0.0375 (12)	0.0573 (14)	0.0106 (10)	0.0263 (12)	0.0054 (10)
C23	0.0521 (14)	0.0381 (12)	0.0375 (12)	0.0097 (11)	0.0052 (11)	−0.0017 (9)
C22	0.0511 (14)	0.0340 (12)	0.0488 (13)	0.0105 (11)	0.0083 (11)	−0.0044 (10)

### 2-(4-Hexyloxyphenyl)-1H-benzimidazole (3) Geometric parameters (Å, º)

**Table:**

O1—C18	1.367 (2)	C10—H10	0.9500
O1—C21	1.439 (2)	C10—C11	1.385 (3)
N2—C7	1.378 (2)	C20—H20	0.9500
N2—C6	1.386 (2)	C16—H16	0.9500
N2—C8	1.458 (2)	C4—H4	0.9500
N1—C7	1.320 (2)	C4—C3	1.402 (3)
N1—C1	1.389 (2)	C3—H3	0.9500
C7—C15	1.469 (2)	C11—H11	0.9500
C9—C8	1.513 (2)	C11—C12	1.379 (3)
C9—C14	1.391 (2)	C13—H13	0.9500
C9—C10	1.390 (2)	C13—C12	1.385 (3)
C6—C1	1.398 (2)	C12—H12	0.9500
C6—C5	1.387 (2)	C21—H21A	0.9900
C15—C20	1.395 (3)	C21—H21B	0.9900
C15—C16	1.386 (3)	C21—C22	1.516 (3)
C1—C2	1.396 (2)	C24—H24A	0.9900
C17—H17	0.9500	C24—H24B	0.9900
C17—C18	1.380 (3)	C24—C25	1.520 (3)
C17—C16	1.389 (2)	C24—C23	1.535 (3)
C18—C19	1.396 (3)	C26—H26A	0.9800
C8—H8A	0.9900	C26—H26B	0.9800
C8—H8B	0.9900	C26—H26C	0.9800
C5—H5	0.9500	C26—C25	1.512 (3)
C5—C4	1.381 (3)	C25—H25A	0.9900
C19—H19	0.9500	C25—H25B	0.9900
C19—C20	1.376 (3)	C23—H23A	0.9900
C2—H2	0.9500	C23—H23B	0.9900
C2—C3	1.382 (3)	C23—C22	1.498 (3)
C14—H14	0.9500	C22—H22A	0.9900
C14—C13	1.384 (3)	C22—H22B	0.9900
			
C18—O1—C21	117.15 (15)	C17—C16—H16	119.4
C7—N2—C6	106.42 (14)	C5—C4—H4	119.2
C7—N2—C8	129.33 (14)	C5—C4—C3	121.69 (17)
C6—N2—C8	124.01 (15)	C3—C4—H4	119.2
C7—N1—C1	105.21 (15)	C2—C3—C4	121.43 (17)
N2—C7—C15	124.45 (16)	C2—C3—H3	119.3
N1—C7—N2	112.75 (15)	C4—C3—H3	119.3
N1—C7—C15	122.76 (16)	C10—C11—H11	119.7
C14—C9—C8	118.62 (15)	C12—C11—C10	120.56 (18)
C10—C9—C8	122.63 (16)	C12—C11—H11	119.7
C10—C9—C14	118.74 (16)	C14—C13—H13	119.8
N2—C6—C1	105.60 (15)	C14—C13—C12	120.37 (18)
N2—C6—C5	131.77 (17)	C12—C13—H13	119.8
C5—C6—C1	122.63 (16)	C11—C12—C13	119.39 (17)
C20—C15—C7	117.75 (16)	C11—C12—H12	120.3
C16—C15—C7	123.98 (16)	C13—C12—H12	120.3
C16—C15—C20	118.20 (16)	O1—C21—H21A	110.2
N1—C1—C6	110.01 (15)	O1—C21—H21B	110.2
N1—C1—C2	129.78 (17)	O1—C21—C22	107.40 (16)
C2—C1—C6	120.21 (16)	H21A—C21—H21B	108.5
C18—C17—H17	120.2	C22—C21—H21A	110.2
C18—C17—C16	119.61 (17)	C22—C21—H21B	110.2
C16—C17—H17	120.2	H24A—C24—H24B	107.6
O1—C18—C17	124.75 (17)	C25—C24—H24A	108.7
O1—C18—C19	115.30 (16)	C25—C24—H24B	108.7
C17—C18—C19	119.95 (17)	C25—C24—C23	114.15 (18)
N2—C8—C9	114.30 (14)	C23—C24—H24A	108.7
N2—C8—H8A	108.7	C23—C24—H24B	108.7
N2—C8—H8B	108.7	H26A—C26—H26B	109.5
C9—C8—H8A	108.7	H26A—C26—H26C	109.5
C9—C8—H8B	108.7	H26B—C26—H26C	109.5
H8A—C8—H8B	107.6	C25—C26—H26A	109.5
C6—C5—H5	121.7	C25—C26—H26B	109.5
C4—C5—C6	116.52 (17)	C25—C26—H26C	109.5
C4—C5—H5	121.7	C24—C25—H25A	109.0
C18—C19—H19	120.2	C24—C25—H25B	109.0
C20—C19—C18	119.69 (17)	C26—C25—C24	112.80 (18)
C20—C19—H19	120.2	C26—C25—H25A	109.0
C1—C2—H2	121.2	C26—C25—H25B	109.0
C3—C2—C1	117.51 (18)	H25A—C25—H25B	107.8
C3—C2—H2	121.2	C24—C23—H23A	109.0
C9—C14—H14	119.7	C24—C23—H23B	109.0
C13—C14—C9	120.51 (17)	H23A—C23—H23B	107.8
C13—C14—H14	119.7	C22—C23—C24	112.89 (17)
C9—C10—H10	119.8	C22—C23—H23A	109.0
C11—C10—C9	120.42 (18)	C22—C23—H23B	109.0
C11—C10—H10	119.8	C21—C22—H22A	108.7
C15—C20—H20	119.4	C21—C22—H22B	108.7
C19—C20—C15	121.19 (17)	C23—C22—C21	114.30 (18)
C19—C20—H20	119.4	C23—C22—H22A	108.7
C15—C16—C17	121.28 (17)	C23—C22—H22B	108.7
C15—C16—H16	119.4	H22A—C22—H22B	107.6
			
O1—C18—C19—C20	−178.63 (16)	C17—C18—C19—C20	1.7 (3)
O1—C21—C22—C23	−60.9 (2)	C18—O1—C21—C22	−171.66 (15)
N2—C7—C15—C20	−141.71 (17)	C18—C17—C16—C15	0.1 (3)
N2—C7—C15—C16	41.3 (3)	C18—C19—C20—C15	0.7 (3)
N2—C6—C1—N1	−0.27 (18)	C8—N2—C7—N1	−174.66 (15)
N2—C6—C1—C2	179.56 (15)	C8—N2—C7—C15	3.3 (3)
N2—C6—C5—C4	179.23 (16)	C8—N2—C6—C1	175.08 (14)
N1—C7—C15—C20	36.1 (2)	C8—N2—C6—C5	−4.0 (3)
N1—C7—C15—C16	−140.91 (18)	C8—C9—C14—C13	179.99 (16)
N1—C1—C2—C3	−178.94 (16)	C8—C9—C10—C11	179.44 (16)
C7—N2—C6—C1	0.31 (17)	C5—C6—C1—N1	178.94 (15)
C7—N2—C6—C5	−178.80 (17)	C5—C6—C1—C2	−1.2 (2)
C7—N2—C8—C9	−105.68 (19)	C5—C4—C3—C2	−0.6 (3)
C7—N1—C1—C6	0.12 (18)	C14—C9—C8—N2	−169.16 (15)
C7—N1—C1—C2	−179.70 (17)	C14—C9—C10—C11	0.1 (3)
C7—C15—C20—C19	−179.81 (16)	C14—C13—C12—C11	−0.1 (3)
C7—C15—C16—C17	179.22 (16)	C10—C9—C8—N2	11.5 (2)
C9—C14—C13—C12	0.6 (3)	C10—C9—C14—C13	−0.6 (3)
C9—C10—C11—C12	0.5 (3)	C10—C11—C12—C13	−0.5 (3)
C6—N2—C7—N1	−0.26 (18)	C20—C15—C16—C17	2.3 (3)
C6—N2—C7—C15	177.70 (15)	C16—C15—C20—C19	−2.7 (3)
C6—N2—C8—C9	80.8 (2)	C16—C17—C18—O1	178.27 (16)
C6—C1—C2—C3	1.3 (2)	C16—C17—C18—C19	−2.1 (3)
C6—C5—C4—C3	0.6 (2)	C21—O1—C18—C17	−5.4 (2)
C1—N1—C7—N2	0.09 (18)	C21—O1—C18—C19	174.93 (16)
C1—N1—C7—C15	−177.91 (15)	C24—C23—C22—C21	−178.67 (18)
C1—C6—C5—C4	0.2 (2)	C25—C24—C23—C22	64.6 (3)
C1—C2—C3—C4	−0.4 (3)	C23—C24—C25—C26	174.14 (18)

### 2-(4-Hexyloxyphenyl)-1-phenyl-1H-benzimidazole (2) Crystal data

**Table:**

C_25_H_26_N_2_O	*F*(000) = 792
*M**_r_* = 370.48	*D*_x_ = 1.209 Mg m^−^^3^
Monoclinic, *P*2_1_/*n*	Mo *K*α radiation, λ = 0.71073 Å
*a* = 9.0089 (3) Å	Cell parameters from 6945 reflections
*b* = 16.6539 (5) Å	θ = 2.5–30.0°
*c* = 13.8400 (5) Å	µ = 0.07 mm^−^^1^
β = 101.419 (1)°	*T* = 100 K
*V* = 2035.36 (12) Å^3^	Block, colourless
*Z* = 4	0.23 × 0.22 × 0.16 mm

### 2-(4-Hexyloxyphenyl)-1-phenyl-1H-benzimidazole (2) Data collection

**Table:**

Bruker D8 Venture diffractometer	6195 independent reflections
Radiation source: microfocus sealed X-ray tube, Incoatec IµS microsource	4489 reflections with *I* > 2σ(*I*)
Focusing mirrors monochromator	*R*_int_ = 0.047
Detector resolution: 10.4 pixels mm^-1^	θ_max_ = 30.5°, θ_min_ = 1.9°
ω–scan	*h* = −12→12
Absorption correction: multi-scan (SADABS; Bruker, 2016)	*k* = −22→23
*T*_min_ = 0.694, *T*_max_ = 0.746	*l* = −19→19
22388 measured reflections	

### 2-(4-Hexyloxyphenyl)-1-phenyl-1H-benzimidazole (2) Refinement

**Table:**

Refinement on *F*^2^	Primary atom site location: dual
Least-squares matrix: full	Hydrogen site location: difference Fourier map
*R*[*F*^2^ > 2σ(*F*^2^)] = 0.047	All H-atom parameters refined
*wR*(*F*^2^) = 0.119	*w* = 1/[σ^2^(*F*_o_^2^) + (0.0568*P*)^2^ + 0.2708*P*] where *P* = (*F*_o_^2^ + 2*F*_c_^2^)/3
*S* = 1.03	(Δ/σ)_max_ = 0.001
6195 reflections	Δρ_max_ = 0.27 e Å^−^^3^
357 parameters	Δρ_min_ = −0.25 e Å^−^^3^
0 restraints	

### 2-(4-Hexyloxyphenyl)-1-phenyl-1H-benzimidazole (2) Special details

**Table:**

Geometry. All esds (except the esd in the dihedral angle between two l.s. planes) are estimated using the full covariance matrix. The cell esds are taken into account individually in the estimation of esds in distances, angles and torsion angles; correlations between esds in cell parameters are only used when they are defined by crystal symmetry. An approximate (isotropic) treatment of cell esds is used for estimating esds involving l.s. planes.

### 2-(4-Hexyloxyphenyl)-1-phenyl-1H-benzimidazole (2) Fractional atomic coordinates and isotropic or equivalent isotropic displacement parameters (Å^2^)

**Table:**

	*x*	*y*	*z*	*U*_iso_*/*U*_eq_	
O1	0.41962 (10)	0.79543 (5)	0.47354 (6)	0.02038 (19)	
N2	0.36688 (10)	0.42504 (6)	0.31526 (7)	0.0158 (2)	
N1	0.59290 (11)	0.46364 (6)	0.28508 (7)	0.0175 (2)	
C1	0.56463 (13)	0.38438 (7)	0.25505 (8)	0.0163 (2)	
C7	0.47394 (12)	0.48562 (7)	0.32094 (8)	0.0153 (2)	
C18	0.36776 (13)	0.65236 (7)	0.48047 (8)	0.0168 (2)	
C14	0.45784 (12)	0.56503 (7)	0.36379 (8)	0.0158 (2)	
C17	0.43040 (13)	0.71868 (7)	0.44203 (9)	0.0166 (2)	
C15	0.52509 (13)	0.63193 (7)	0.32834 (9)	0.0177 (2)	
C8	0.22065 (12)	0.42800 (7)	0.34137 (8)	0.0155 (2)	
C6	0.42416 (13)	0.35920 (7)	0.27312 (8)	0.0162 (2)	
C19	0.38161 (13)	0.57656 (7)	0.44118 (8)	0.0160 (2)	
C16	0.51118 (13)	0.70773 (7)	0.36634 (9)	0.0180 (2)	
C9	0.19533 (13)	0.38329 (7)	0.42090 (9)	0.0192 (2)	
C5	0.36509 (14)	0.28298 (7)	0.24870 (9)	0.0201 (2)	
C11	−0.05825 (14)	0.43579 (7)	0.39489 (9)	0.0215 (3)	
C2	0.65337 (14)	0.33068 (7)	0.21309 (9)	0.0201 (2)	
C13	0.10770 (13)	0.47566 (7)	0.28723 (9)	0.0200 (2)	
C4	0.45431 (14)	0.23105 (8)	0.20649 (9)	0.0218 (3)	
C3	0.59663 (14)	0.25456 (8)	0.18966 (9)	0.0216 (3)	
C10	0.05474 (14)	0.38751 (8)	0.44755 (9)	0.0224 (3)	
C12	−0.03216 (14)	0.47956 (8)	0.31463 (9)	0.0224 (3)	
C20	0.32560 (15)	0.80774 (7)	0.54534 (9)	0.0211 (2)	
C23	0.23511 (16)	1.03530 (7)	0.50281 (9)	0.0234 (3)	
C21	0.32315 (16)	0.89670 (8)	0.56663 (9)	0.0237 (3)	
C22	0.24677 (16)	0.94655 (8)	0.47849 (9)	0.0244 (3)	
C24	0.15475 (17)	1.08422 (8)	0.41511 (10)	0.0268 (3)	
C25	0.1401 (2)	1.17278 (8)	0.43819 (11)	0.0314 (3)	
H15	0.5791 (15)	0.6253 (8)	0.2753 (10)	0.020 (3)*	
H20A	0.2200 (16)	0.7877 (8)	0.5168 (10)	0.020 (3)*	
H18	0.3139 (15)	0.6582 (8)	0.5347 (10)	0.017 (3)*	
H10	0.0330 (17)	0.3558 (9)	0.5043 (12)	0.033 (4)*	
H19	0.3366 (14)	0.5308 (8)	0.4683 (10)	0.016 (3)*	
H20B	0.3691 (16)	0.7754 (8)	0.6061 (11)	0.025 (4)*	
H21A	0.2665 (18)	0.9038 (9)	0.6220 (12)	0.032 (4)*	
H22A	0.3029 (18)	0.9409 (9)	0.4231 (13)	0.039 (4)*	
H23A	0.3409 (17)	1.0592 (8)	0.5256 (11)	0.024 (4)*	
H9	0.2784 (16)	0.3505 (8)	0.4602 (11)	0.025 (4)*	
H12	−0.1128 (17)	0.5134 (9)	0.2784 (12)	0.030 (4)*	
H4	0.4210 (16)	0.1761 (9)	0.1892 (11)	0.028 (4)*	
H13	0.1299 (15)	0.5066 (8)	0.2297 (11)	0.025 (4)*	
H22B	0.1433 (19)	0.9249 (9)	0.4523 (12)	0.034 (4)*	
H23B	0.1808 (17)	1.0410 (9)	0.5594 (12)	0.031 (4)*	
H2	0.7493 (17)	0.3472 (9)	0.1987 (11)	0.029 (4)*	
H21B	0.4285 (17)	0.9151 (8)	0.5899 (11)	0.029 (4)*	
H5	0.2680 (16)	0.2683 (8)	0.2613 (10)	0.018 (3)*	
H24A	0.0477 (19)	1.0608 (9)	0.3914 (12)	0.040 (5)*	
H24B	0.209 (2)	1.0783 (9)	0.3595 (14)	0.044 (5)*	
H3	0.6594 (16)	0.2171 (8)	0.1605 (11)	0.023 (4)*	
H11	−0.1567 (16)	0.4405 (8)	0.4156 (10)	0.023 (4)*	
H16	0.5570 (16)	0.7553 (9)	0.3410 (11)	0.026 (4)*	
H25A	0.0885 (17)	1.2038 (9)	0.3793 (12)	0.032 (4)*	
H25B	0.0814 (19)	1.1804 (10)	0.4929 (13)	0.045 (5)*	
H25C	0.243 (2)	1.1968 (11)	0.4611 (14)	0.054 (5)*	

### 2-(4-Hexyloxyphenyl)-1-phenyl-1H-benzimidazole (2) Atomic displacement parameters (Å^2^)

**Table:**

	*U*^11^	*U*^22^	*U*^33^	*U*^12^	*U*^13^	*U*^23^
O1	0.0227 (4)	0.0154 (4)	0.0239 (4)	0.0001 (3)	0.0067 (3)	−0.0025 (3)
N2	0.0119 (4)	0.0160 (5)	0.0196 (5)	0.0006 (4)	0.0032 (4)	−0.0006 (4)
N1	0.0150 (5)	0.0185 (5)	0.0191 (5)	0.0017 (4)	0.0040 (4)	0.0006 (4)
C1	0.0153 (5)	0.0174 (5)	0.0157 (5)	0.0021 (4)	0.0020 (4)	0.0011 (4)
C7	0.0130 (5)	0.0167 (5)	0.0159 (5)	0.0007 (4)	0.0019 (4)	0.0026 (4)
C18	0.0150 (5)	0.0190 (6)	0.0164 (5)	0.0014 (4)	0.0030 (4)	0.0004 (4)
C14	0.0116 (5)	0.0176 (5)	0.0175 (5)	0.0018 (4)	0.0010 (4)	0.0009 (4)
C17	0.0143 (5)	0.0154 (5)	0.0189 (5)	0.0015 (4)	0.0003 (4)	0.0001 (4)
C15	0.0138 (5)	0.0213 (6)	0.0183 (5)	0.0005 (4)	0.0044 (4)	0.0012 (4)
C8	0.0118 (5)	0.0162 (5)	0.0191 (5)	−0.0011 (4)	0.0044 (4)	−0.0017 (4)
C6	0.0142 (5)	0.0179 (5)	0.0159 (5)	0.0026 (4)	0.0019 (4)	0.0000 (4)
C19	0.0136 (5)	0.0169 (5)	0.0170 (5)	0.0005 (4)	0.0019 (4)	0.0023 (4)
C16	0.0151 (5)	0.0180 (6)	0.0209 (6)	−0.0026 (4)	0.0033 (4)	0.0018 (4)
C9	0.0160 (5)	0.0183 (6)	0.0225 (6)	0.0013 (5)	0.0022 (4)	0.0037 (4)
C5	0.0177 (6)	0.0218 (6)	0.0199 (6)	−0.0017 (5)	0.0015 (5)	−0.0015 (4)
C11	0.0143 (5)	0.0252 (6)	0.0264 (6)	−0.0009 (5)	0.0076 (5)	0.0007 (5)
C2	0.0173 (6)	0.0244 (6)	0.0191 (6)	0.0032 (5)	0.0051 (4)	0.0007 (5)
C13	0.0165 (5)	0.0241 (6)	0.0197 (6)	0.0010 (5)	0.0043 (4)	0.0048 (5)
C4	0.0242 (6)	0.0196 (6)	0.0199 (6)	0.0000 (5)	0.0004 (5)	−0.0034 (5)
C3	0.0237 (6)	0.0220 (6)	0.0188 (6)	0.0057 (5)	0.0037 (5)	−0.0033 (5)
C10	0.0208 (6)	0.0236 (6)	0.0241 (6)	−0.0017 (5)	0.0080 (5)	0.0054 (5)
C12	0.0148 (5)	0.0260 (6)	0.0259 (6)	0.0045 (5)	0.0029 (5)	0.0052 (5)
C20	0.0261 (6)	0.0192 (6)	0.0183 (6)	0.0028 (5)	0.0051 (5)	−0.0007 (5)
C23	0.0310 (7)	0.0183 (6)	0.0207 (6)	0.0017 (5)	0.0048 (5)	−0.0027 (5)
C21	0.0318 (7)	0.0201 (6)	0.0182 (6)	0.0025 (5)	0.0028 (5)	−0.0034 (5)
C22	0.0336 (7)	0.0192 (6)	0.0193 (6)	0.0038 (5)	0.0029 (5)	−0.0027 (5)
C24	0.0387 (8)	0.0204 (6)	0.0212 (6)	0.0044 (6)	0.0055 (6)	−0.0018 (5)
C25	0.0467 (9)	0.0193 (6)	0.0274 (7)	0.0037 (6)	0.0048 (6)	−0.0001 (5)

### 2-(4-Hexyloxyphenyl)-1-phenyl-1H-benzimidazole (2) Geometric parameters (Å, º)

**Table:**

O1—C17	1.3602 (13)	C11—H11	0.988 (14)
O1—C20	1.4423 (14)	C2—C3	1.3812 (18)
N2—C7	1.3872 (14)	C2—H2	0.964 (15)
N2—C8	1.4342 (14)	C13—C12	1.3874 (16)
N2—C6	1.3884 (14)	C13—H13	1.002 (15)
N1—C1	1.3921 (15)	C4—C3	1.4034 (18)
N1—C7	1.3189 (14)	C4—H4	0.978 (14)
C1—C6	1.4019 (16)	C3—H3	0.981 (14)
C1—C2	1.4000 (16)	C10—H10	0.997 (15)
C7—C14	1.4683 (15)	C12—H12	0.976 (15)
C18—C17	1.3930 (16)	C20—C21	1.5115 (17)
C18—C19	1.3899 (16)	C20—H20A	1.011 (14)
C18—H18	0.976 (13)	C20—H20B	1.010 (14)
C14—C15	1.4025 (16)	C23—C22	1.5241 (17)
C14—C19	1.3954 (16)	C23—C24	1.5209 (18)
C17—C16	1.4007 (16)	C23—H23A	1.023 (15)
C15—C16	1.3826 (16)	C23—H23B	1.007 (16)
C15—H15	0.964 (14)	C21—C22	1.5226 (18)
C8—C9	1.3847 (16)	C21—H21A	1.008 (16)
C8—C13	1.3874 (16)	C21—H21B	0.989 (15)
C6—C5	1.3920 (17)	C22—H22A	1.002 (17)
C19—H19	0.972 (13)	C22—H22B	0.999 (17)
C16—H16	0.989 (15)	C24—C25	1.5204 (18)
C9—C10	1.3893 (17)	C24—H24A	1.032 (17)
C9—H9	0.996 (14)	C24—H24B	0.998 (18)
C5—C4	1.3861 (17)	C25—H25A	0.999 (16)
C5—H5	0.957 (14)	C25—H25B	1.014 (18)
C11—C10	1.3857 (17)	C25—H25C	1.000 (19)
C11—C12	1.3869 (17)		
			
C17—O1—C20	116.33 (9)	C12—C13—H13	121.9 (8)
C7—N2—C8	128.59 (9)	C5—C4—C3	121.40 (12)
C7—N2—C6	106.62 (9)	C5—C4—H4	120.8 (8)
C6—N2—C8	124.70 (9)	C3—C4—H4	117.8 (8)
C7—N1—C1	105.14 (9)	C2—C3—C4	121.66 (11)
N1—C1—C6	110.40 (9)	C2—C3—H3	117.6 (8)
N1—C1—C2	130.08 (11)	C4—C3—H3	120.7 (8)
C2—C1—C6	119.52 (11)	C9—C10—H10	120.9 (9)
N2—C7—C14	123.70 (10)	C11—C10—C9	120.20 (11)
N1—C7—N2	112.61 (10)	C11—C10—H10	118.9 (9)
N1—C7—C14	123.69 (10)	C11—C12—C13	120.09 (11)
C17—C18—H18	121.0 (8)	C11—C12—H12	119.2 (9)
C19—C18—C17	119.75 (10)	C13—C12—H12	120.7 (9)
C19—C18—H18	119.2 (8)	O1—C20—C21	107.87 (10)
C15—C14—C7	119.35 (10)	O1—C20—H20A	108.6 (8)
C19—C14—C7	122.48 (10)	O1—C20—H20B	108.3 (8)
C19—C14—C15	118.14 (10)	C21—C20—H20A	110.5 (8)
O1—C17—C18	124.23 (10)	C21—C20—H20B	112.2 (8)
O1—C17—C16	116.29 (10)	H20A—C20—H20B	109.2 (11)
C18—C17—C16	119.48 (10)	C22—C23—H23A	110.2 (8)
C14—C15—H15	119.5 (8)	C22—C23—H23B	109.2 (8)
C16—C15—C14	120.95 (11)	C24—C23—C22	112.71 (11)
C16—C15—H15	119.5 (8)	C24—C23—H23A	107.7 (8)
C9—C8—N2	119.07 (10)	C24—C23—H23B	110.0 (9)
C9—C8—C13	121.22 (10)	H23A—C23—H23B	106.8 (12)
C13—C8—N2	119.71 (10)	C20—C21—C22	113.57 (11)
N2—C6—C1	105.22 (9)	C20—C21—H21A	107.0 (8)
N2—C6—C5	131.77 (11)	C20—C21—H21B	108.5 (8)
C5—C6—C1	123.00 (10)	C22—C21—H21A	109.2 (9)
C18—C19—C14	121.42 (11)	C22—C21—H21B	110.2 (9)
C18—C19—H19	119.0 (8)	H21A—C21—H21B	108.3 (12)
C14—C19—H19	119.6 (8)	C23—C22—H22A	109.1 (9)
C17—C16—H16	118.4 (8)	C23—C22—H22B	109.3 (9)
C15—C16—C17	120.20 (11)	C21—C22—C23	113.07 (11)
C15—C16—H16	121.4 (8)	C21—C22—H22A	110.2 (9)
C8—C9—C10	119.09 (11)	C21—C22—H22B	109.6 (9)
C8—C9—H9	120.3 (8)	H22A—C22—H22B	105.3 (13)
C10—C9—H9	120.6 (8)	C23—C24—H24A	108.6 (9)
C6—C5—H5	120.6 (8)	C23—C24—H24B	109.5 (10)
C4—C5—C6	116.46 (11)	C25—C24—C23	113.54 (11)
C4—C5—H5	123.0 (8)	C25—C24—H24A	108.6 (9)
C10—C11—C12	120.19 (11)	C25—C24—H24B	109.5 (9)
C10—C11—H11	120.1 (8)	H24A—C24—H24B	106.9 (13)
C12—C11—H11	119.7 (8)	C24—C25—H25A	112.1 (9)
C1—C2—H2	120.8 (9)	C24—C25—H25B	111.0 (9)
C3—C2—C1	117.94 (11)	C24—C25—H25C	110.0 (10)
C3—C2—H2	121.2 (9)	H25A—C25—H25B	108.5 (13)
C8—C13—H13	118.9 (8)	H25A—C25—H25C	107.6 (13)
C12—C13—C8	119.19 (11)	H25B—C25—H25C	107.5 (14)
			
O1—C17—C16—C15	−178.63 (10)	C15—C14—C19—C18	2.07 (17)
O1—C20—C21—C22	65.39 (14)	C8—N2—C7—N1	−176.10 (10)
N2—C7—C14—C15	−150.27 (11)	C8—N2—C7—C14	4.92 (18)
N2—C7—C14—C19	31.78 (17)	C8—N2—C6—C1	176.60 (10)
N2—C8—C9—C10	178.61 (11)	C8—N2—C6—C5	−2.10 (19)
N2—C8—C13—C12	−178.46 (11)	C8—C9—C10—C11	0.10 (19)
N2—C6—C5—C4	179.78 (12)	C8—C13—C12—C11	−0.39 (19)
N1—C1—C6—N2	−0.11 (12)	C6—N2—C7—N1	0.61 (13)
N1—C1—C6—C5	178.73 (11)	C6—N2—C7—C14	−178.37 (10)
N1—C1—C2—C3	−179.72 (12)	C6—N2—C8—C9	71.01 (15)
N1—C7—C14—C15	30.87 (16)	C6—N2—C8—C13	−109.35 (13)
N1—C7—C14—C19	−147.09 (11)	C6—C1—C2—C3	0.98 (17)
C1—N1—C7—N2	−0.66 (13)	C6—C5—C4—C3	0.08 (18)
C1—N1—C7—C14	178.32 (10)	C19—C18—C17—O1	178.21 (10)
C1—C6—C5—C4	1.28 (18)	C19—C18—C17—C16	−2.17 (17)
C1—C2—C3—C4	0.32 (18)	C19—C14—C15—C16	−2.51 (17)
C7—N2—C8—C9	−112.83 (13)	C9—C8—C13—C12	1.17 (18)
C7—N2—C8—C13	66.81 (16)	C5—C4—C3—C2	−0.88 (19)
C7—N2—C6—C1	−0.28 (12)	C2—C1—C6—N2	179.31 (10)
C7—N2—C6—C5	−178.97 (12)	C2—C1—C6—C5	−1.85 (18)
C7—N1—C1—C6	0.47 (13)	C13—C8—C9—C10	−1.03 (18)
C7—N1—C1—C2	−178.88 (12)	C10—C11—C12—C13	−0.5 (2)
C7—C14—C15—C16	179.44 (11)	C12—C11—C10—C9	0.7 (2)
C7—C14—C19—C18	−179.95 (10)	C20—O1—C17—C18	−5.57 (16)
C18—C17—C16—C15	1.73 (17)	C20—O1—C17—C16	174.80 (10)
C14—C15—C16—C17	0.65 (18)	C20—C21—C22—C23	175.95 (12)
C17—O1—C20—C21	−177.40 (10)	C22—C23—C24—C25	179.25 (13)
C17—C18—C19—C14	0.26 (17)	C24—C23—C22—C21	−178.39 (12)

### 1-Benzyl-2-(4-hexyloxyphenyl)-1H-benzimidazole (1) Crystal data

**Table:**

C_19_H_22_N_2_O	*D*_x_ = 1.190 Mg m^−^^3^
*M**_r_* = 294.38	Mo *K*α radiation, λ = 0.71073 Å
Orthorhombic, *P**b**c**a*	Cell parameters from 3935 reflections
*a* = 9.3802 (13) Å	θ = 2.2–22.2°
*b* = 9.4076 (13) Å	µ = 0.07 mm^−^^1^
*c* = 37.235 (5) Å	*T* = 150 K
*V* = 3285.8 (8) Å^3^	Plate, colourless
*Z* = 8	0.16 × 0.14 × 0.01 mm
*F*(000) = 1264	

### 1-Benzyl-2-(4-hexyloxyphenyl)-1H-benzimidazole (1) Data collection

**Table:**

Bruker D8 Venture diffractometer	2900 independent reflections
Radiation source: microfocus sealed X-ray tube, Incoatec IµS microsource	2171 reflections with *I* > 2σ(*I*)
Focusing mirrors monochromator	*R*_int_ = 0.097
Detector resolution: 10.4 pixels mm^-1^	θ_max_ = 25.1°, θ_min_ = 2.2°
ω–scan	*h* = −11→11
Absorption correction: multi-scan (SADABS; Bruker, 2016)	*k* = −11→11
*T*_min_ = 0.642, *T*_max_ = 0.745	*l* = −44→44
26780 measured reflections	

### 1-Benzyl-2-(4-hexyloxyphenyl)-1H-benzimidazole (1) Refinement

**Table:**

Refinement on *F*^2^	Primary atom site location: dual
Least-squares matrix: full	Hydrogen site location: inferred from neighbouring sites
*R*[*F*^2^ > 2σ(*F*^2^)] = 0.099	H-atom parameters constrained
*wR*(*F*^2^) = 0.233	*w* = 1/[σ^2^(*F*_o_^2^) + (0.0273*P*)^2^ + 15.0864*P*] where *P* = (*F*_o_^2^ + 2*F*_c_^2^)/3
*S* = 1.17	(Δ/σ)_max_ < 0.001
2900 reflections	Δρ_max_ = 0.32 e Å^−^^3^
200 parameters	Δρ_min_ = −0.40 e Å^−^^3^
0 restraints	

### 1-Benzyl-2-(4-hexyloxyphenyl)-1H-benzimidazole (1) Special details

**Table:**

Geometry. All esds (except the esd in the dihedral angle between two l.s. planes) are estimated using the full covariance matrix. The cell esds are taken into account individually in the estimation of esds in distances, angles and torsion angles; correlations between esds in cell parameters are only used when they are defined by crystal symmetry. An approximate (isotropic) treatment of cell esds is used for estimating esds involving l.s. planes.

### 1-Benzyl-2-(4-hexyloxyphenyl)-1H-benzimidazole (1) Fractional atomic coordinates and isotropic or equivalent isotropic displacement parameters (Å^2^)

**Table:**

	*x*	*y*	*z*	*U*_iso_*/*U*_eq_	
O1	0.7545 (4)	0.5030 (4)	0.59420 (9)	0.0414 (9)	
N2	0.2163 (4)	0.2653 (4)	0.68379 (10)	0.0248 (9)	
H2	0.268160	0.187574	0.682290	0.030*	
N1	0.1515 (4)	0.4926 (4)	0.67692 (10)	0.0272 (9)	
C7	0.2513 (5)	0.3952 (5)	0.67041 (11)	0.0244 (10)	
C6	0.0860 (5)	0.2772 (5)	0.69998 (12)	0.0229 (10)	
C1	0.0453 (5)	0.4199 (5)	0.69573 (12)	0.0244 (10)	
C8	0.3840 (5)	0.4218 (5)	0.65126 (12)	0.0237 (10)	
C11	0.6376 (5)	0.4715 (5)	0.61439 (13)	0.0311 (11)	
C2	−0.0840 (5)	0.4681 (5)	0.70942 (12)	0.0298 (11)	
H2A	−0.113329	0.564031	0.706417	0.036*	
C4	−0.1271 (5)	0.2283 (5)	0.73179 (12)	0.0299 (11)	
H4	−0.187766	0.164485	0.744326	0.036*	
C9	0.5082 (5)	0.3519 (5)	0.66103 (13)	0.0300 (11)	
H9	0.506235	0.285528	0.680252	0.036*	
C5	−0.0007 (5)	0.1807 (5)	0.71803 (12)	0.0292 (11)	
H5	0.027284	0.084213	0.720724	0.035*	
C13	0.3884 (5)	0.5194 (5)	0.62292 (12)	0.0300 (11)	
H13	0.304450	0.569382	0.616270	0.036*	
C3	−0.1679 (5)	0.3716 (5)	0.72747 (12)	0.0306 (11)	
H3	−0.256049	0.402423	0.737335	0.037*	
C12	0.5135 (5)	0.5432 (5)	0.60466 (13)	0.0318 (11)	
H12	0.515327	0.608955	0.585293	0.038*	
C10	0.6359 (5)	0.3777 (5)	0.64308 (13)	0.0334 (12)	
H10	0.720987	0.331308	0.650433	0.040*	
C16	1.0202 (6)	0.6351 (6)	0.57073 (14)	0.0403 (13)	
H16A	1.064798	0.675292	0.592588	0.048*	
H16B	0.927145	0.682857	0.567265	0.048*	
C15	0.9959 (6)	0.4787 (6)	0.57613 (15)	0.0423 (13)	
H15A	1.087936	0.433602	0.582430	0.051*	
H15B	0.963661	0.436941	0.553110	0.051*	
C14	0.8880 (5)	0.4424 (7)	0.60488 (15)	0.0448 (14)	
H14A	0.879059	0.337954	0.607315	0.054*	
H14B	0.918389	0.482004	0.628292	0.054*	
C17	1.1158 (6)	0.6661 (6)	0.53834 (16)	0.0497 (15)	
H17A	1.213683	0.633557	0.543928	0.060*	
H17B	1.081204	0.609693	0.517656	0.060*	
C18	1.1219 (7)	0.8192 (7)	0.52750 (17)	0.0563 (17)	
H18A	1.161793	0.875489	0.547597	0.068*	
H18B	1.023846	0.853648	0.522954	0.068*	
C19	1.2111 (8)	0.8441 (8)	0.49442 (17)	0.072 (2)	
H19A	1.176104	0.784013	0.474815	0.108*	
H19B	1.310699	0.820335	0.499581	0.108*	
H19C	1.204377	0.944261	0.487356	0.108*	

### 1-Benzyl-2-(4-hexyloxyphenyl)-1H-benzimidazole (1) Atomic displacement parameters (Å^2^)

**Table:**

	*U*^11^	*U*^22^	*U*^33^	*U*^12^	*U*^13^	*U*^23^
O1	0.0270 (18)	0.055 (2)	0.0419 (19)	−0.0024 (17)	0.0043 (16)	0.0105 (18)
N2	0.021 (2)	0.0145 (18)	0.039 (2)	−0.0004 (15)	0.0016 (16)	0.0028 (16)
N1	0.025 (2)	0.0174 (19)	0.040 (2)	0.0004 (16)	−0.0001 (18)	0.0023 (17)
C7	0.025 (2)	0.016 (2)	0.033 (2)	−0.0009 (18)	−0.003 (2)	−0.0003 (18)
C6	0.018 (2)	0.019 (2)	0.031 (2)	−0.0008 (18)	−0.0023 (19)	−0.0022 (19)
C1	0.022 (2)	0.018 (2)	0.033 (2)	−0.0010 (19)	0.000 (2)	0.0018 (19)
C8	0.022 (2)	0.016 (2)	0.033 (2)	−0.0036 (18)	0.0011 (19)	−0.0003 (19)
C11	0.024 (2)	0.033 (3)	0.036 (3)	−0.003 (2)	0.001 (2)	−0.001 (2)
C2	0.027 (2)	0.020 (2)	0.042 (3)	0.003 (2)	0.003 (2)	−0.001 (2)
C4	0.031 (3)	0.023 (2)	0.036 (3)	−0.006 (2)	0.004 (2)	0.000 (2)
C9	0.028 (3)	0.023 (2)	0.038 (3)	0.000 (2)	0.000 (2)	0.006 (2)
C5	0.030 (3)	0.019 (2)	0.039 (3)	−0.004 (2)	−0.002 (2)	0.002 (2)
C13	0.025 (3)	0.027 (3)	0.038 (3)	−0.002 (2)	−0.004 (2)	0.002 (2)
C3	0.019 (2)	0.032 (3)	0.041 (3)	−0.002 (2)	0.005 (2)	−0.003 (2)
C12	0.030 (3)	0.026 (3)	0.039 (3)	−0.004 (2)	−0.003 (2)	0.008 (2)
C10	0.025 (3)	0.035 (3)	0.041 (3)	0.000 (2)	0.000 (2)	0.003 (2)
C16	0.028 (3)	0.043 (3)	0.050 (3)	0.001 (2)	0.008 (2)	−0.001 (3)
C15	0.033 (3)	0.043 (3)	0.051 (3)	0.000 (3)	0.013 (3)	−0.003 (3)
C14	0.025 (3)	0.053 (4)	0.056 (3)	0.002 (3)	0.007 (2)	0.011 (3)
C17	0.046 (4)	0.048 (4)	0.055 (3)	−0.001 (3)	0.013 (3)	−0.002 (3)
C18	0.053 (4)	0.047 (4)	0.068 (4)	−0.011 (3)	0.016 (3)	−0.007 (3)
C19	0.083 (5)	0.072 (5)	0.061 (4)	−0.030 (4)	0.016 (4)	−0.003 (4)

### 1-Benzyl-2-(4-hexyloxyphenyl)-1H-benzimidazole (1) Geometric parameters (Å, º)

**Table:**

O1—C11	1.362 (6)	C13—H13	0.9500
O1—C14	1.433 (6)	C13—C12	1.375 (7)
N2—H2	0.8800	C3—H3	0.9500
N2—C7	1.360 (6)	C12—H12	0.9500
N2—C6	1.368 (5)	C10—H10	0.9500
N1—C7	1.333 (6)	C16—H16A	0.9900
N1—C1	1.397 (6)	C16—H16B	0.9900
C7—C8	1.456 (6)	C16—C15	1.503 (8)
C6—C1	1.405 (6)	C16—C17	1.531 (7)
C6—C5	1.392 (6)	C15—H15A	0.9900
C1—C2	1.392 (6)	C15—H15B	0.9900
C8—C9	1.387 (6)	C15—C14	1.512 (7)
C8—C13	1.399 (6)	C14—H14A	0.9900
C11—C12	1.394 (7)	C14—H14B	0.9900
C11—C10	1.386 (7)	C17—H17A	0.9900
C2—H2A	0.9500	C17—H17B	0.9900
C2—C3	1.377 (6)	C17—C18	1.497 (8)
C4—H4	0.9500	C18—H18A	0.9900
C4—C5	1.367 (7)	C18—H18B	0.9900
C4—C3	1.410 (7)	C18—C19	1.507 (8)
C9—H9	0.9500	C19—H19A	0.9800
C9—C10	1.392 (7)	C19—H19B	0.9800
C5—H5	0.9500	C19—H19C	0.9800
			
C11—O1—C14	117.7 (4)	C13—C12—H12	119.8
C7—N2—H2	126.1	C11—C10—C9	119.4 (5)
C7—N2—C6	107.7 (4)	C11—C10—H10	120.3
C6—N2—H2	126.1	C9—C10—H10	120.3
C7—N1—C1	104.8 (4)	H16A—C16—H16B	107.9
N2—C7—C8	122.7 (4)	C15—C16—H16A	109.1
N1—C7—N2	112.4 (4)	C15—C16—H16B	109.1
N1—C7—C8	124.9 (4)	C15—C16—C17	112.4 (5)
N2—C6—C1	105.7 (4)	C17—C16—H16A	109.1
N2—C6—C5	133.0 (4)	C17—C16—H16B	109.1
C5—C6—C1	121.3 (4)	C16—C15—H15A	108.6
N1—C1—C6	109.3 (4)	C16—C15—H15B	108.6
C2—C1—N1	130.2 (4)	C16—C15—C14	114.7 (5)
C2—C1—C6	120.5 (4)	H15A—C15—H15B	107.6
C9—C8—C7	120.5 (4)	C14—C15—H15A	108.6
C9—C8—C13	119.0 (4)	C14—C15—H15B	108.6
C13—C8—C7	120.5 (4)	O1—C14—C15	107.4 (4)
O1—C11—C12	115.1 (4)	O1—C14—H14A	110.2
O1—C11—C10	125.0 (4)	O1—C14—H14B	110.2
C10—C11—C12	119.9 (4)	C15—C14—H14A	110.2
C1—C2—H2A	121.2	C15—C14—H14B	110.2
C3—C2—C1	117.5 (4)	H14A—C14—H14B	108.5
C3—C2—H2A	121.2	C16—C17—H17A	108.6
C5—C4—H4	119.8	C16—C17—H17B	108.6
C5—C4—C3	120.4 (4)	H17A—C17—H17B	107.6
C3—C4—H4	119.8	C18—C17—C16	114.7 (5)
C8—C9—H9	119.5	C18—C17—H17A	108.6
C8—C9—C10	120.9 (4)	C18—C17—H17B	108.6
C10—C9—H9	119.5	C17—C18—H18A	109.0
C6—C5—H5	120.9	C17—C18—H18B	109.0
C4—C5—C6	118.3 (4)	C17—C18—C19	113.0 (5)
C4—C5—H5	120.9	H18A—C18—H18B	107.8
C8—C13—H13	119.8	C19—C18—H18A	109.0
C12—C13—C8	120.3 (4)	C19—C18—H18B	109.0
C12—C13—H13	119.8	C18—C19—H19A	109.5
C2—C3—C4	122.0 (4)	C18—C19—H19B	109.5
C2—C3—H3	119.0	C18—C19—H19C	109.5
C4—C3—H3	119.0	H19A—C19—H19B	109.5
C11—C12—H12	119.8	H19A—C19—H19C	109.5
C13—C12—C11	120.4 (4)	H19B—C19—H19C	109.5
			
O1—C11—C12—C13	179.5 (4)	C1—N1—C7—C8	−180.0 (4)
O1—C11—C10—C9	−178.4 (5)	C1—C6—C5—C4	−0.3 (7)
N2—C7—C8—C9	−35.0 (7)	C1—C2—C3—C4	−0.9 (7)
N2—C7—C8—C13	145.6 (4)	C8—C9—C10—C11	−1.8 (7)
N2—C6—C1—N1	0.1 (5)	C8—C13—C12—C11	−0.6 (7)
N2—C6—C1—C2	−179.9 (4)	C11—O1—C14—C15	173.5 (4)
N2—C6—C5—C4	179.1 (5)	C9—C8—C13—C12	1.3 (7)
N1—C7—C8—C9	145.0 (5)	C5—C6—C1—N1	179.7 (4)
N1—C7—C8—C13	−34.4 (7)	C5—C6—C1—C2	−0.4 (7)
N1—C1—C2—C3	−179.2 (4)	C5—C4—C3—C2	0.2 (7)
C7—N2—C6—C1	−0.2 (5)	C13—C8—C9—C10	0.0 (7)
C7—N2—C6—C5	−179.6 (5)	C3—C4—C5—C6	0.3 (7)
C7—N1—C1—C6	−0.1 (5)	C12—C11—C10—C9	2.5 (7)
C7—N1—C1—C2	180.0 (5)	C10—C11—C12—C13	−1.3 (8)
C7—C8—C9—C10	−179.4 (4)	C16—C15—C14—O1	61.1 (6)
C7—C8—C13—C12	−179.4 (4)	C16—C17—C18—C19	−177.1 (5)
C6—N2—C7—N1	0.1 (5)	C15—C16—C17—C18	169.2 (5)
C6—N2—C7—C8	−179.9 (4)	C14—O1—C11—C12	174.6 (5)
C6—C1—C2—C3	0.9 (7)	C14—O1—C11—C10	−4.6 (7)
C1—N1—C7—N2	0.0 (5)	C17—C16—C15—C14	−172.9 (5)

### 1-Benzyl-2-(4-hexyloxyphenyl)-1H-benzimidazole (1) Hydrogen-bond geometry (Å, º)

**Table:**

*D*—H···*A*	*D*—H	H···*A*	*D*···*A*	*D*—H···*A*
N2—H2···N1^i^	0.88	1.99	2.861 (5)	169

Symmetry code: (i) −*x*+1/2, *y*−1/2, *z*.
